# Radiological Evaluation of the Accuracy of Demirjian, Nolla, and Willems Methods for Dental Age Estimation in 3–17-Year-Old Iranian Children

**DOI:** 10.1155/2024/8783660

**Published:** 2024-07-03

**Authors:** Benjamin Pliska, Azam Nahvi, Nikta Pakdaman, Sepideh Dadgar, Mehdi Aryana, Farhad Sobouti

**Affiliations:** ^1^ Division of Orthodontics Department of Oral Health Sciences Faculty of Dentistry University of British Columbia, Vancouver, British Columbia, Canada; ^2^ Dental Research Center Mazandaran University of Medical Sciences, Sari, Iran; ^3^ Department of Pediatric Dentistry Faculty of Dentistry Mazandaran University of Medical Sciences, Sari, Iran; ^4^ Student Research Committee Faculty of Dentistry Mazandaran University of Medical Sciences, Sari, Iran; ^5^ Department of Orthodontics Faculty of Dentistry Mazandaran University of Medical Sciences, Sari, Iran; ^6^ Orthodontics DSATP Candidate Faculty of Dentistry University of Toronto, Toronto, Canada

**Keywords:** Age determination by teeth, chronological age, Demirjian method, dental maturity, diagnostic methods, forensic dentistry, growth and development, Nolla method, panoramic radiography, Willems method

## Abstract

**Background:** The stage of tooth formation is one of the most reliable indicators for predicting a patient's developmental age by radiographs. This study compared the accuracy of three distinct dental age estimation methods (Demirjian, Nolla, and Willems) in children aged 3–17 in the northern Iranian population.

**Methods:** This cross-sectional study examined panoramic radiographs of 434 children aged 3–17 from Mazandaran Province, Iran, who had teeth 31–37 present on the left mandible. This study employed the Demirjian, Nolla, and Willems methods to estimate the dental age of the sample and compare it with the chronological age. The data were analyzed using SPSS v16. A paired *t*-test was used to compare chronological and dental ages. The Pearson correlation was used to correlate the chronological and dental ages. The errors of different methods were compared using the Wilcoxon test. *P* values < 0.05 were considered significant for all tests except Wilcoxon. For Wilcoxon, a *P* value < 0.017 was considered significant.

**Results:** The three methods presented differing mean estimated ages. The Demirjian method delivered the highest mean, and all three methods differed significantly when compared in pairs. The results showed that the Demirjian method overestimated chronological age by 0.25 years (*P* < 0.001) in girls and 0.09 years (*P* = 0.28) in boys. The Willems method underestimated chronological age by 0.05 years (*P* = 0.47) in girls and 0.12 years (*P* = 0.13) in boys. The Nolla method underestimated chronological age by 0.41 years (*P* < 0.001) in girls and 0.40 years (*P* < 0.001) in boys. The accuracy of each method varied with the patient's age.

**Conclusion:** According to the findings, the Willems method outperformed the Demirjian method, and the Demirjian method exceeded the Nolla method for estimating dental age in Iranian children aged 3–17. Overall, the Demirjian method overestimated the age of the study population, whereas the other two underestimated it.

## 1. Introduction

Age determination is a crucial step in the information gathering of several dental procedures, such as pediatric dentistry, orthodontics, and medical treatments, including endocrinology, forensic medicine, and disease detection [1]. Chronological age estimation allows the dentist to evaluate the progress and determine the most appropriate treatment for different dental malocclusions based on the children's craniofacial development [2, 3]. There are several age estimation indices, such as the third molar maturity index, and various methods, such as general physical examination, molecular biomarkers (e.g., DNA methylation), multiphoton microscopy, left-handed radiographic images, and dental images [4–7]. In order to increase the accuracy of age estimation and improve diagnostic standards, the field of forensic age diagnosis recommends that an X-ray examination of the left hand, a dental growth assessment, and physical examination tests be performed in a combined manner [8]. However, a consensus on the best method for predicting chronological age remains elusive [9].

Currently, one of the most common methods for estimating chronological age is calculating dental age through the stages of tooth mineralization. Several methods have been introduced and tested in different populations [10]. Nolla's method was introduced in 1960 [11]. There is a high degree of agreement (more than 90%) among dentists in the clinical field regarding this dental age estimation method; however, it still has to be tested in different populations [10, 12]. In 1973, Demirjian, Goldstein, and Tanner studied a sample of French-Canadian children. They presented a method for age estimation based on eight developmental stages in the first seven teeth of the lower jaw [13]. This method was combined and updated with two other age estimation methods based on four teeth by Demirjian and Goldstein in 1976 [14]. The Demirjian method is one of the most popular tools for estimating chronological age due to its simplicity, consensus among clinicians, and ease of standardization and reproducibility [15]. This method was also investigated in the Iranian 3–9-year-old population in Isfahan, and its accuracy was sufficient for the study population [1]. The Willems method is a modification of the Demirjian method, published in 1973 [13, 16]. It has been used for different populations, presenting a smaller age estimate than other methods and being more accurate in some populations [17, 18]. However, this method showed to be in need of more research as it is affected by racial diversities [19, 20].

The estimation of age through tooth mineralization is a well known and acceptable method for orthodontists because it shows less diversity than other skeletal or sexual growth characteristics. However, the hereditary, functional, environmental, gender, nutritional, and metabolic factors must be considered when applying these methods, as they can vary among differing populations. Therefore, evaluating the accuracy and efficiency of age estimation methods for different populations is essential [10]. Additionally, although the Demirjian method was evaluated in several studies on Iranian children and adolescents [1, 21–26], only a few studies examined other methods [27–29] and no studies compared the Demirjian, Nolla, and Willems methods comprehensively. Therefore, this study is aimed at examining and comparing the accuracy of three dental age estimation methods of Demirjian, Nolla, and Willems in 3–17-year-old children in Mazandaran Province, Iran.

## 2. Materials and Methods

In this cross-sectional study, panoramic radiographs (available in the archives of the Oromaxillofacial Radiology Department from 2018 to 2021) belonging to children aged 3–17 who visited the dental clinic of Mazandaran University of Medical Sciences were collected. Then, an oral and maxillofacial radiologist reviewed each radiograph for eligibility. The methods of Han et al. were followed in this study [5]. For the analysis, panoramic radiographs of each patient with seven permanent teeth present on the left side of the mandible (teeth 31–37) were included in the study. The exclusion criteria encompassed panoramic radiographs in which the age and gender were not recorded or had improper resolution, leading to the wrong assessment and diagnosis of the degree of dental growth and development. Furthermore, children with systemic diseases such as syndromes or changes in tooth growth and development, extracted permanent teeth (except the third molar), orthodontic appliances, and a history of dental trauma were excluded. In considering sample size, for a confidence interval of 95% and a power of 90%, a minimum of 434 images was required for this study [9].

The panoramic radiographs were obtained using a digital panoramic scanner (Planmeca ProMax 2D, Helsinki, Finland), with a minimum exposure time of 16 s, a voltage of 66 kV, and a current of 9 mA. The panoramic images were saved in JPEG format. The eligible panoramic radiographs were analyzed in random order, and the clinical history number, date of birth, date of imaging, and gender were recorded. The degree of tooth calcification and dental age of each child was estimated using the three different methods of Willems, Nolla, and Demirjian based on the study conducted by Cortés et al. [9].

Chronological age was calculated by subtracting the date of radiography from the date of birth. Dental age was obtained based on the degree of dental growth using the three methods. Then, chronological age was subtracted from dental age; a positive result indicated overestimation, while a negative one showed underestimation.

- The Demirjian method evaluates the degree of development of each of the left mandibular teeth (except the third molar) by classifying them on an 8-step scale indicated by the letters “A” (as the lowest degree of mineralization) to “H” (as the highest degree of mineralization). The stage marked by the letters is converted into a grade by gender using a conversion table provided by Demirjian, Goldstein, and Tanner [13]. All the numerical scores are added, and the result is converted into dental age by referring to another table regarding gender [13].
- The Nolla method evaluates the degree of dental development of the left mandibular teeth (except the third molar) by assigning each of them a stage between 1 (no sign of calcification) and 10 (apical end completed). If the tooth was between stages, an appropriate fraction (0.2, 0.5, or 0.7) was added as recommended by Nolla. The sum of the scores is then converted to the dental age based on the conversion tables provided by Nolla for each gender [11].
- The Willems method evaluates the degree of growth of each of the left mandibular teeth (except the third molar) using the A-to-H classification method proposed by Demirjian. A score is assigned to each of the seven teeth, which is converted into an average score concerning gender in a calculation by Willems et al. [16]. The sum of the values will show the dental age.

**Figure 1:**
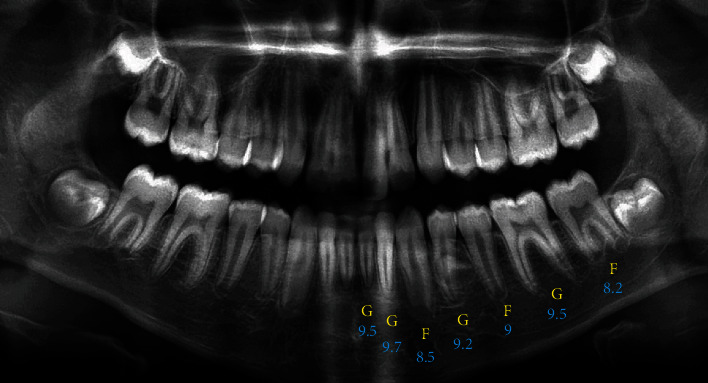
The scoring of the teeth on a panoramic radiograph according to the Demirjian (yellow), Nolla (blue), and Willems (yellow) methods.

Figure 1 shows an example of the scoring of the teeth on the panoramic radiograph of an 11-year-old boy according to each of the three methods. Based on the conversion tables mentioned by Demirjian, Goldstein, and Tanner [13], Nolla [11], and Willems et al. [16], the scorings estimated the age of the boy as 9.3, 10.9, and 10.7 years old, respectively.

The dental age estimation in all of the radiographs was performed by two orthodontists, where 204 panoramic radiographs were analyzed by one and the other 230 by another. In order to measure the interobserver agreement, 75 radiographs were selected randomly, and their dental age was estimated by the two observers using all three methods of Demirjian, Nolla, and Willems. Kappa statistics were calculated to assess the interobserver agreement. In addition, each observer performed the measurements on the same set of radiographs after 2 weeks. The intraclass correlation coefficient (ICC) was used to assess the intraobserver reliability.

### 2.1. Statistical Analysis

Data were analyzed with SPSS v16. A paired *t*-test was utilized for the comparison of chronological and dental age. The Wilcoxon test was used for the comparison of the errors of three methods. The Pearson correlation was used to correlate the chronological and dental age. A *P* value < 0.05 was considered significant for all tests except Wilcoxon. For Wilcoxon, a *P* value < 0.017 was considered significant.

## 3. Results

The records of a total of 462 patients were initially screened, and after the removal of images of low quality, lacking sufficient information, or cases of congenital tooth agenesis, 434 panoramic radiographs were available for analysis. Of the 434 patients, 190 were boys (43.8%) and 244 were girls (56.2%). The interobserver agreement in all three methods and the total showed a high correlation between the two observers, and excellent reliability was established (Table 1). In addition, an excellent degree of intraobserver reliability was found for both of the observers in all three methods (Table 1).

The results of this study showed that the Demirjian method overestimated chronological age by 0.25 years (*P* < 0.001) for girls and 0.09 years (*P* = 0.28) for boys (Table 2). Comparisons between dental age and chronological age for the Demirjian method in each age category for both genders are presented in Table 3. The Demirjian method overestimated the dental age for both genders in all age groups, except for girls aged 4, 10, 12, and 16 and boys aged 3, 9, 10, 11, 12, and 16.

The Willems method underestimated chronological age by 0.05 years (*P* = 0.47) for girls and 0.12 years (*P* = 0.13) for boys (Table 2). Comparisons between dental age and chronological age for the Willems method in each age category for both genders are presented in Table 4. The Willems method underestimated the dental age for both genders in all age groups, except for girls aged 3, 6, 10, 13, 14, and 15 and boys aged 4, 7, 14, and 15.

The Nolla method overestimated chronological age by 0.41 years (*P* < 0.001) for girls and 0.40 years (*P* < 0.001) for boys (Table 2). Comparisons between dental age and chronological age for the Nolla method in each age category for both genders are presented in Table 5. The Nolla method overestimated the dental age for both genders in all age groups, except for girls aged 3, 6, 13, and 15 and boys aged 3, 4, 5, 7, and 15.

**Figure 2:**
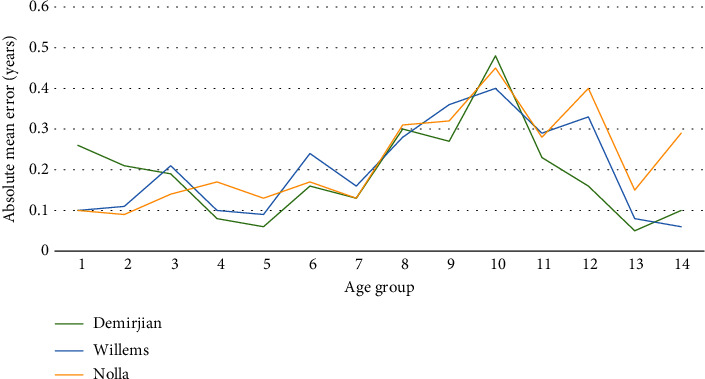
Comparison of the absolute mean error among the Demirjian, Willems, and Nolla methods for girls.

**Figure 3:**
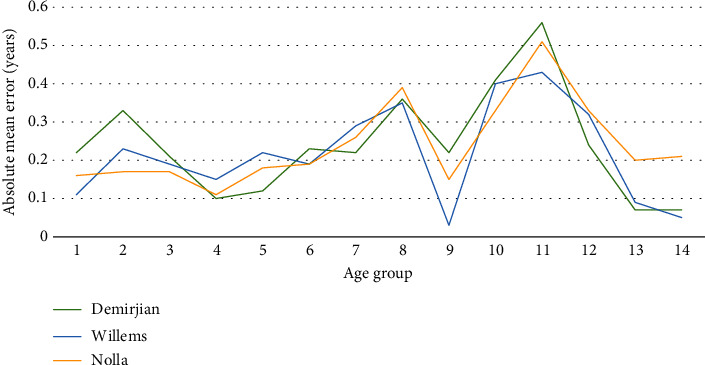
Comparison of the absolute mean error among the Demirjian, Willems, and Nolla methods for boys.

The differences in absolute mean error among the three methods are shown in Table 2. Figures 2 and 3 show that the absolute mean error was lowest for the Demirjian method in girls and the Willems method in boys and highest for the Demirjian method in boys.

The results of the Pearson correlations showed that all three methods of Demirjian, Willems, and Nolla were correlated in both genders (*P* < 0.001) (Table 6).

Regarding the general difference between the three methods, Table 7 reports the twosome comparison using the Wilcoxon test and reveals that all three methods are significantly different (*P* < 0.001). A comparison of the Demirjian and Willems methods demonstrates a significant difference between the two in the age groups of 5, 6, 7, and 9 years for girls and the age groups of 3, 4, 5, and 6 years for boys. In general, there is a significant difference between the two methods regarding gender. Moreover, there is a significant difference between the Willems and Nolla methods in the age groups of 7, 10, 11, 13, and 14 years for girls, as well as the age groups of 5, 7, and 14 years for boys. Finally, a significant difference exists between the Demirjian and Nolla methods in all age groups of girls except for the age group of 3, 4, 10, 12, and 15 years and also in all age groups of boys except for 8, 9, 10, 11, 15, and 16 years.

## 4. Discussion

Determining patient age is essential for collecting information in daily clinical practice and decision-making for treatment planning [30, 31]. Age estimation is also useful in a variety of legal situations, such as those involving immigration or misreported ages on documentation. Of all the techniques for determining dental age, including histological, morphological, biochemical, and radiological techniques, the radiological technique is the least invasive and the most simple and repeatable one [32, 33]. Numerous radiographic techniques have been created and investigated to examine dental mineralization as a measure of age [10]. The most critical issue in dental age estimation is the accuracy of the applied methods. Due to the effect of different environmental factors, eating habits, growth rate, and ethnicity on the timing of tooth formation, the accuracy of dental age estimation methods should be investigated in different populations [34, 35]. Since the investigation of Demirjian, Willems, and Nolla methods in diverse populations has presented different results, this study explored these three methods of dental age estimation in a population of 3–17-year-old children in Mazandaran Province, Iran.

To date, various studies have tested the accuracy of dental age estimation methods in different regions. The Demirjian method is considered one of the most straightforward and practical methods for age estimation, although its reliability is questionable for some populations [5, 22]. Many studies have shown that this method overestimates the dental age in different populations [22, 36]. In line with previous studies, the findings of this study demonstrated that the mean estimated age was higher than the mean chronological age of the study children using the Demirjian method.

Consistent with this study, in 2020, Han et al. studied 1000 Chinese boys and 1000 girls and reported that the Demirjian method overestimated children's ages [5]. A meta-analysis study by Yan et al., in which 26 studies were selected after reviewing 370 articles using the Demirjian method with a total of 11,499 children, showed that the mean age estimated by this method in Asian and Caucasian races was greater than the chronological age [36]. Another meta-analysis by Jayaraman et al., with 34 selected articles out of 274 articles published between 1973 and 2011, displayed that the mean age estimated by the Demirjian method in the French-Canadian race was higher than the chronological age [37]. The results of both meta-analyses were consistent with the findings of the present study regarding the Demirjian method.

The Willems method has only one conversion table for each gender, in which the amount of tooth growth is calculated and the dental age is calculated directly from the sum of the scores. Using a conversion table improves efficiency by avoiding long steps in the calculation. Several studies, including those of Han et al. [5]; Galić et al. [38]; El-Bakary, Hammad, and Mohammed [39]; and Willems et al. [16], have shown that the Willems method overestimates children's age compared to their chronological age. Contrary to the results of the above studies, the present study revealed that the dental age of the Willems method was estimated to be lower than the children's chronological age. The reason for this difference can be seen in the difference in race, sample size, and population age range. Han et al. studied 2000 Chinese children aged 5–14 [5]; Galić et al. studied 1089 Bosnian and Herzegovinian children aged 6–13 [38]; El-Bakary, Hammad, and Mohammed conducted their study on 72 Egyptian children with an age range of 5–16 [39]; and Willems et al. examined 2116 Belgian children with an age range of 1.8–18 [16], while this study investigated 434 Iranian children with an age range of 3–17. Consistent with the results of this research, Jun reported that the average age calculated by the Willems method was lower than the average chronological age in a study in 2005 in China [40].

The Nolla method was one of the first to evaluate tooth formation longitudinally. Javadi Nejad et al. [29] and Miloglu et al. [12] reported that the dental age was lower than the chronological age in different populations. Consistent with these studies, the results of the current study showed that the mean dental age was lower than their mean chronological age, using the Nolla method in all 434 children examined. However, overestimation has also been reported for the Nolla method in Malaysian and South Indian populations [41, 42].

In some age and gender subgroups of this study, the estimated age value in the three methods of Demirjian, Nolla, and Willems had a significant difference. Han et al. investigated three methods (Demirjian, Nolla, and Willems) in Chinese children aged 5–15. In contrast to the present study, they found that the difference between dental age and chronological age in Demirjian's method was significant in all age and gender subgroups. However, the difference between dental age and chronological age was significant in all age groups of boys except 10-year-olds and all age groups of girls except 7-, 9-, and 10-year-olds in the Willems method. On the other hand, the difference between dental and chronological age was significant only in the age groups of 5- and 14-year-olds for boys and age groups of 6-, 9-, 13-, and 14-year-olds for girls in the Nolla method [5]. Djukic et al.'s study on 686 Serbian children aged 4–15 showed that in the boys aged 5, 6, and 12–15 and all age groups of girls (except 4-, 9-, and 10-year-olds), there was a significant difference between dental and chronological age for both the Demirjian and Willems methods [43].

In the paired comparison of Demirjian and Willems methods, we found a significant difference in the 5-, 6-, 7-, and 9-year-olds for girls, as well as in the 3-, 4-, 5-, and 6-year-olds for boys. Mani et al. reviewed 428 children aged 7–15 in Malaysia and observed a significant difference between dental age and chronological age for Demirjian and Willems methods in most age groups [44]. The studies of Djukic et al. on 686 Serbian children aged 4–15 [43]; Urzel and Bruzek on 743 French children aged 4–15 [45]; Cameriere et al. on 756 Croatian, Italian, and Spanish children aged 5–15 [20]; and Medina and Blanco on 238 Venezuelan children aged 5–13 [46] showed that the accuracy of the Willems method was higher than the Demirjian method.

In the paired comparison of Willems and Nolla methods in this study, a significant difference was observed between the two methods in the 7-, 10-, 11-, 13-, and 14-year-olds for girls, as well as in the 5-, 7-, and 14-year-olds for boys. Hegde, Patodia, and Dixit studied 1200 Indian children aged 5–16. Regarding the Willems method, there was a significant difference between dental age and chronological age in the age groups of 8, 10, and 15 years for boys and 6, 11, 13, and 14 years for girls. However, there was a significant difference between dental age and chronological age in the 7-, 9-, 11-, 12-, and 13-year-old boys and the 7-, 8-, 10-, 12-, and 14-year-old girls using the Nolla method. Furthermore, the difference between the accuracy of the Willems and Nolla methods in age estimation was statistically significant [3].

On the other hand, in the direct comparison of the Demirjian and Nolla methods, a significant difference was observed between the two methods in all age groups of girls except for the age group of 3, 4, 10, 12, and 15 years and also in all age groups of boys except for 9, 10, 11, 15, and 16 years. Several studies have compared the Demirjian and Nolla methods, and all found that the Nolla method was more accurate than the Demirjian. For example, in Nur et al.'s study, 630 panoramic radiographs of children aged 5–15.9 in northern Turkey were examined. The difference between dental and chronological age for the Demirjian method was 0.86 years and 0.54 years for the Nolla method [47]. Kirzioglu, Ceyhan, and Bayraktar in a study on 425 Turkish children aged 7–13 [48], Melo and Ata-Ali on 2641 Spanish individuals aged 7–21 [49], Tomas et al. on 821 Spanish and Portuguese individuals aged 4–34 [50], and Mohammed et al. on 660 children aged 6–16 in South India [42] reported that the Nolla method was more accurate than the Demirjian method in both genders. However, the opposite result was recorded, and the Demirjian method was more accurate than the Nolla in the current study.

When it comes to patients in the developmental age group, forensic medicine accepts an error range of 0.5–1.0 years between the patient's estimated dental age and chronological age [51]. The findings of this study showed that the Willems method met these criteria better than the other two methods. The results of the present study revealed that the Demirjian method overestimated the age (the mean dental age was more than the mean chronological age) and the Willems and Nolla methods underestimated the age (the mean dental age was lower than that of the chronological age). Furthermore, among these three methods, the Willems and Nolla methods showed the most and the least accuracy, respectively. In line with this study, Cortés et al. reported that the Willems method was more accurate than the other two methods [9]. Correspondingly, Kelmendi et al. examined the Demirjian, Chaillet, and Willems methods. They reported that the Willems method showed the slightest difference between dental and chronological age in 5–14-year-old Kosovar children [52]. Mani et al. presented that the accuracy of the Willems method was higher than the Demirjian method [44]. Unlike these studies, Han et al. observed that the Nolla method was the most accurate and the Demirjian method was the least accurate [5]. These differences can be due to the differences in race and age range of the study population. On the other hand, environmental factors such as nutrition, eating habits, and lifestyle, which significantly impact the growth of teeth, vary in different regions. Another reason is the difference in sample size, which may affect the accuracy of dental age estimation.

This study was subjected to some limitations, including a lack of access to a larger sample size during the study period and an unbalanced distribution of samples across age and gender groups. These factors may have an impact on the generalizability of the findings in some study groups. Therefore, it is recommended that future research considers a larger sample size, populations of diverse races, and an equal distribution of samples by age and gender.

## 5. Conclusions

The Willems technique of age estimation was more accurate than the Demirjian method, and the Demirjian method was more accurate than the Nolla method in Iranian children aged 3–17. Additionally, it was discovered that the Demirjian method overestimates age in the population under study, while the other two methods underestimate it.

## Figures and Tables

**Table 1:** The inter- and intraobserver agreement results.

**Method**	**Interobserver agreement**	**Intraobserver agreement**	
	**Kappa value**	**Observer**	**ICC**
Demirjian	0.985	1	0.998
		2	0.996
Willems	0.988	1	0.998
		2	0.995
Nolla	0.989	1	0.994
		2	0.993
Total	0.987	—	—

**Table 2:** Accuracy comparison of the Demirjian, Willems, and Nolla methods of dental age estimation.

**Gender**	**N**	**Method**	**Mean (SD)**		**Paired differences**				**P** ** value**
			**Chronological age (years)**	**Dental age (years)**	**Mean age difference (years)**	**Absolute mean error (years)**	**95% CI of age difference**		
							**Lower**	**Upper**	
Girl	244	Demirjian	9.95 (3.94)	10.19 (4.06)	−0.25	0.06	−0.37	−0.12	< 0.001^∗^
		Willems		9.90 (4.05)	0.05	0.07	−0.08	0.18	0.47
		Nolla		9.54 (3.96)	0.41	0.07	0.27	0.55	< 0.001^∗^

Boy	190	Demirjian	9.93 (4.01)	10.02 (3.97)	−0.09	0.09	−0.26	0.08	0.28
		Willems		9.81 (4.04)	0.12	0.08	−0.04	0.27	0.13
		Nolla		9.53 (3.87)	0.40	0.08	0.23	0.56	< 0.001^∗^

^*^Paired *t*-test, *P* < 0.05 statistically significant.

**Table 3:** Comparison between chronological age and dental age of the Demirjian method in each age category.

**Gender**	**Age group (years)**	**N**	**Mean (SD)**		**Paired differences**				**P** ** value**
			**Chronological age (years)**	**Dental age (years)**	**Mean age difference (years)**	**Absolute mean error (years)**	**95% CI of age difference**		
							**Lower**	**Upper**	
Girl	3.00–3.99	12	3.55 (0.25)	3.76 (1.02)	−0.22	0.26	−0.79	0.36	0.422
	4.00–4.99	20	4.38 (0.28)	4.37 (1.00)	0.01	0.21	−0.43	0.45	0.953
	5.00–5.99	21	5.47 (0.31)	5.98 (0.84)	−0.51	0.19	−0.90	−0.11	0.015^∗^
	6.00–6.99	12	6.56 (0.33)	7.27 (0.17)	−0.71	0.08	−0.89	−0.53	< 0.001^∗^
	7.00–7.99	24	7.51 (0.22)	7.76 (0.29)	−0.25	0.06	−0.37	−0.12	< 0.001^∗^
	8.00–8.99	16	8.42 (0.29)	8.51 (0.72)	−0.09	0.16	−0.43	0.25	0.569
	9.00–9.99	14	9.46 (0.26)	9.71 (0.54)	−0.25	0.13	−0.53	0.03	0.074
	10.00–10.99	23	10.45 (0.29)	10.33 (1.44)	0.11	0.30	−0.50	0.73	0.704
	11.00–11.99	21	11.29 (0.19)	11.67 (1.21)	−0.38	0.27	−0.94	0.18	0.171
	12.00–12.99	14	12.51 (0.29)	11.84 (1.87)	0.67	0.48	−0.37	1.71	0.185
	13.00–13.99	14	13.35 (0.30)	14.08 (0.84)	−0.73	0.23	−1.22	−0.23	0.007^∗^
	14.00–14.99	18	14.47 (0.30)	15.51 (0.71)	−1.04	0.16	−1.38	−0.70	< 0.001^∗^
	15.00–15.99	22	15.57 (0.25)	16.00 (0.02)	−0.42	0.05	−0.53	−0.31	< 0.001^∗^
	16.00–16.99	13	16.43 (0.22)	15.92 (0.28)	0.51	0.10	0.30	0.72	< 0.001^∗^

Boy	3.00–3.99	10	3.54 (0.28)	2.89 (0.52)	0.65	0.22	0.16	1.14	0.015^∗^
	4.00–4.99	13	4.41 (0.31)	5.29 (1.11)	−0.88	0.33	−1.59	−0.16	0.020^∗^
	5.00–5.99	13	5.41 (0.33)	6.27 (0.79)	−0.86	0.21	−1.31	−0.41	0.001^∗^
	6.00–6.99	18	6.35 (0.26)	6.99 (0.44)	−0.65	0.10	−0.85	−0.44	< 0.001^∗^
	7.00–7.99	14	7.40 (0.29)	8.06 (0.62)	−0.66	0.12	−0.91	−0.41	< 0.001^∗^
	8.00–8.99	12	8.38 (0.24)	8.57 (0.91)	−0.20	0.23	−0.71	0.31	0.414
	9.00–9.99	20	9.28 (0.23)	9.00 (0.96)	0.28	0.22	−0.19	0.75	0.229
	10.00–10.99	20	10.53 (0.27)	10.37 (1.64)	0.15	0.36	−0.60	0.90	0.679
	11.00–11.99	6	11.40 (0.22)	10.43 (0.39)	0.98	0.22	0.41	1.54	0.007^∗^
	12.00–12.99	13	12.53 (0.28)	11.23 (1.63)	1.30	0.41	0.41	2.19	0.008^∗^
	13.00–13.99	11	13.43 (0.36)	13.73 (1.63)	−0.29	0.56	−1.55	0.96	0.612
	14.00–14.99	8	14.59 (0.23)	15.66 (0.65)	−1.07	0.24	−1.63	−0.52	0.003^∗^
	15.00–15.99	13	15.44 (0.29)	15.95 (0.14)	−0.52	0.07	−0.66	−0.37	< 0.001^∗^
	16.00–16.99	19	16.42 (0.22)	15.95 (0.23)	0.48	0.07	0.32	0.63	< 0.001^∗^

^*^Paired *t*-test, *P* < 0.05 statistically significant.

**Table 4:** Comparison between chronological age and dental age of the Willems method in each age category.

**Gender**	**Age group (years)**	**N**	**Mean (SD)**		**Paired differences**				**P** ** value**
			**Chronological age (years)**	**Dental age (years)**	**Mean age difference (years)**	**Absolute mean error (years)**	**95% CI of age difference**		
							**Lower**	**Upper**	
Girl	3.00–3.99	12	3.55 (0.25)	4.16 (0.30)	−0.61	0.10	−0.83	−0.40	< 0.001^∗^
	4.00–4.99	20	4.38 (0.28)	4.27 (0.48)	0.12	0.11	−0.11	0.34	0.291
	5.00–5.99	21	5.47 (0.31)	5.34 (0.86)	0.14	0.21	−0.30	0.58	0.514
	6.00–6.99	12	6.56 (0.33)	6.71 (0.50)	−0.15	0.10	−0.37	0.06	0.145
	7.00–7.99	24	7.51 (0.22)	7.45 (0.54)	0.07	0.09	−0.11	0.25	0.453
	8.00–8.99	16	8.42 (0.29)	8.18 (0.92)	0.24	0.24	−0.27	0.75	0.334
	9.00–9.99	14	9.46 (0.26)	9.12 (0.67)	0.34	0.16	−0.01	0.70	0.058
	10.00–10.99	23	10.45 (0.29)	10.48 (1.32)	−0.04	0.28	−0.62	0.54	0.896
	11.00–11.99	21	11.29 (0.19)	11.22 (1.70)	0.07	0.36	−0.68	0.82	0.848
	12.00–12.99	14	12.51 (0.29)	11.40 (1.59)	1.10	0.40	0.25	1.96	0.015^∗^
	13.00–13.99	14	13.35 (0.30)	13.67 (1.11)	−0.32	0.29	−0.93	0.30	0.289
	14.00–14.99	18	14.47 (0.30)	15.00 (1.48)	−0.53	0.33	−1.23	0.18	0.132
	15.00–15.99	22	15.57 (0.25)	15.76 (0.35)	−0.18	0.08	−0.34	-0.03	0.024^∗^
	16.00–16.99	13	16.43 (0.22)	15.87 (0.12)	0.57	0.06	0.43	0.71	< 0.001^∗^

Boy	3.00–3.99	10	3.54 (0.28)	3.52 (0.18)	0.02	0.11	−0.22	0.26	0.877
	4.00–4.99	13	4.41 (0.31)	4.68 (0.87)	−0.27	0.23	−0.76	0.23	0.261
	5.00–5.99	13	5.41 (0.33)	5.37 (0.73)	0.05	0.19	−0.36	0.45	0.810
	6.00–6.99	18	6.35 (0.26)	6.27 (0.67)	0.07	0.15	−0.25	0.39	0.643
	7.00–7.99	14	7.40 (0.29)	7.87 (1.01)	−0.46	0.22	−0.93	0.01	0.052
	8.00–8.99	12	8.38 (0.24)	8.32 (0.79)	0.06	0.19	−0.37	0.48	0.775
	9.00–9.99	20	9.28 (0.23)	9.13 (1.19)	0.15	0.29	−0.46	0.76	0.613
	10.00–10.99	20	10.53 (0.27)	10.44 (1.62)	0.09	0.35	−0.65	0.83	0.807
	11.00–11.99	6	11.40 (0.22)	10.38 (0.15)	1.03	0.03	0.94	1.12	< 0.001^∗^
	12.00–12.99	13	12.53 (0.28)	10.98 (1.62)	1.55	0.40	0.67	2.42	0.002^∗^
	13.00–13.99	11	13.43 (0.36)	13.24 (1.30)	0.19	0.43	−0.77	1.15	0.666
	14.00–14.99	8	14.59 (0.23)	15.51 (0.92)	−0.92	0.32	−1.67	−0.18	0.022^∗^
	15.00–15.99	13	15.44 (0.29)	15.81 (0.25)	−0.37	0.09	−0.58	−0.17	0.002^∗^
	16.00–16.99	19	16.42 (0.22)	15.96 (0.11)	0.46	0.05	0.35	0.58	< 0.001^∗^

^*^Paired *t*-test, *P* < 0.05 statistically significant.

**Table 5:** Comparison between chronological age and dental age of the Nolla method in each age category.

**Gender**	**Age group (years)**	**N**	**Mean (SD)**		**Paired differences**				**P** ** value**
			**Chronological age (years)**	**Dental age (years)**	**Mean age difference (years)**	**Absolute mean error (years)**	**95% CI of age difference**		
							**Lower**	**Upper**	
Girl	3.00–3.99	12	3.55 (0.25)	3.99 (0.31)	−0.44	0.10	−0.68	−0.21	0.001^∗^
	4.00–4.99	20	4.38 (0.28)	4.22 (0.39)	0.16	0.09	−0.02	0.35	0.083
	5.00–5.99	21	5.47 (0.31)	5.07 (0.68)	0.40	0.14	0.11	0.69	0.010^∗^
	6.00–6.99	12	6.56 (0.33)	6.58 (0.70)	−0.02	0.17	−0.40	0.35	0.901
	7.00–7.99	24	7.51 (0.22)	7.08 (0.60)	0.43	0.13	0.17	0.70	0.003^∗^
	8.00–8.99	16	8.42 (0.29)	7.75 (0.69)	0.67	0.17	0.30	1.04	0.001^∗^
	9.00–9.99	14	9.46 (0.26)	9.05 (0.45)	0.41	0.13	0.13	0.68	0.007^∗^
	10.00–10.99	23	10.45 (0.29)	9.99 (1.46)	0.45	0.31	−0.18	1.09	0.153
	11.00–11.99	21	11.29 (0.19)	10.39 (1.54)	0.90	0.32	0.22	1.57	0.012^∗^
	12.00–12.99	14	12.51 (0.29)	11.33 (1.79)	1.18	0.45	0.20	2.16	0.022^∗^
	13.00–13.99	14	13.35 (0.30)	13.37 (1.09)	−0.02	0.28	−0.62	0.57	0.939
	14.00–14.99	18	14.47 (0.30)	14.03 (1.76)	0.44	0.40	−0.40	1.28	0.285
	15.00–15.99	22	15.57 (0.25)	15.70 (0.71)	−0.12	0.15	−0.44	0.19	0.431
	16.00–16.99	13	16.43 (0.22)	15.23 (0.93)	1.20	0.29	0.58	1.83	0.001^∗^

Boy	3.00–3.99	10	3.54 (0.28)	3.69 (0.34)	−0.15	0.16	−0.52	0.21	0.375
	4.00–4.99	13	4.41 (0.31)	4.64 (0.65)	−0.23	0.17	−0.59	0.14	0.201
	5.00–5.99	13	5.41 (0.33)	5.47 (0.65)	−0.06	0.17	−0.42	0.31	0.743
	6.00–6.99	18	6.35 (0.26)	6.16 (0.48)	0.19	0.11	−0.05	0.43	0.108
	7.00–7.99	14	7.40 (0.29)	7.48 (0.85)	−0.08	0.18	−0.47	0.30	0.650
	8.00–8.99	12	8.38 (0.24)	8.25 (0.76)	0.13	0.19	−0.29	0.55	0.509
	9.00–9.99	20	9.28 (0.23)	8.96 (1.08)	0.32	0.26	−0.23	0.86	0.236
	10.00–10.99	20	10.53 (0.27)	10.03 (1.77)	0.50	0.39	−0.31	1.31	0.210
	11.00–11.99	6	11.40 (0.22)	10.01 (0.34)	1.40	0.15	1.02	1.77	< 0.001^∗^
	12.00–12.99	13	12.53 (0.28)	10.28 (1.31)	2.24	0.33	1.53	2.96	< 0.001^∗^
	13.00–13.99	11	13.43 (0.36)	12.96 (1.56)	0.48	0.51	−0.66	1.61	0.372
	14.00–14.99	8	14.59 (0.23)	14.39 (0.98)	0.21	0.33	−0.57	0.98	0.550
	15.00–15.99	13	15.44 (0.29)	15.61 (0.69)	−0.17	0.20	−0.60	0.27	0.414
	16.00–16.99	19	16.42 (0.22)	15.48 (0.96)	0.94	0.21	0.50	1.39	< 0.001^∗^

^*^Paired *t*-test, *P* < 0.05 statistically significant.

**Table 6:** The correlations among the three methods in both genders.

**Dental age estimation method**	**Pearson correlation (** **P** **value)**	
	**Girl (** **n** = 244**)**	**Boy (** **n** = 190**)**
Demirjian	0.970 (< 0.001^∗^)	0.957 (< 0.001^∗^)
Willems	0.967 (< 0.001^∗^)	0.964 (< 0.001^∗^)
Nolla	0.961 (< 0.001^∗^)	0.957 (< 0.001^∗^)

^*^Pearson correlation, *P* < 0.05 statistically significant.

**Table 7:** Paired comparison of the methods by gender and age using.

**Gender**	**Age**	**P** ** value** ^∗^		
		**Demirjian and Willems**	**Willems and Nolla**	**Demirjian and Nolla**
Girl (*n* = 244)	3.00–3.99	0.114	0.018	0.394
	4.00–4.99	0.531	0.508	0.349
	5.00–5.99	0.001^∗^	0.118	< 0.001^∗^
	6.00–6.99	0.001^∗^	0.176	0.004^∗^
	7.00–7.99	0.001^∗^	0.002^∗^	< 0.001^∗^
	8.00–8.99	0.086	0.055	< 0.001^∗^
	9.00–9.99	< 0.001^∗^	0.571	< 0.001^∗^
	10.00–10.99	0.394	0.013^∗^	0.114
	11.00–11.99	0.034	< 0.001^∗^	< 0.001^∗^
	12.00–12.99	0.080	0.724	0.028
	13.00–13.99	0.103	0.016^∗^	0.002^∗^
	14.00–14.99	0.40	< 0.001^∗^	< 0.001^∗^
	15.00–15.99	0.04	0.512	0.063
	16.00–16.99	0.358	0.022	0.006^∗^
	Total	< 0.001^∗^	< 0.001^∗^	< 0.001^∗^

Boy (*n* = 190)	3.00–3.99	0.002^∗^	0.067	< 0.001^∗^
	4.00–4.99	0.003^∗^	0.650	0.004^∗^
	5.00–5.99	< 0.001^∗^	0.014^∗^	< 0.001^∗^
	6.00–6.99	< 0.001^∗^	0.223	< 0.001^∗^
	7.00–7.99	0.120	0.002^∗^	< 0.001^∗^
	8.00–8.99	0.048	0.358	0.037
	9.00–9.99	0.472	0.130	0.831
	10.00–10.99	0.683	0.018	0.129
	11.00–11.99	0.806	0.050	0.203
	12.00–12.99	0.250	0.018	0.006^∗^
	13.00–13.99	0.138	0.094	0.008^∗^
	14.00–14.99	0.216	0.005^∗^	0.002^∗^
	15.00–15.99	0.059	0.174	0.095
	16.00–16.99	0.772	0.033	0.024
	Total	< 0.001^∗^	< 0.001^∗^	< 0.001^∗^

^*^Wilcoxon test, *P* < 0.017 statistically significant.

## Data Availability

The datasets used and/or analyzed during the current study are available from the corresponding author upon reasonable request.
